# Financing Maternal and Child Health—What Are the Limitations in Estimating Donor Flows and Resource Needs?

**DOI:** 10.1371/journal.pmed.1000305

**Published:** 2010-07-06

**Authors:** Marco Schäferhoff, Christina Schrade, Gavin Yamey

**Affiliations:** 1 Evidence to Policy initiative, SEEK Development, Berlin, Germany; 2 Evidence to Policy initiative, Global Health Group, University of California, San Francisco, San Francisco, California, United States of America

## Abstract

Marco Schäferhoff and colleagues critique funding estimates for the maternal and child health Millennium Development Goals, and make recommendations for improving the tracking of financing flows and estimating the costs of scaling up interventions for mothers and children.

**Summary Points**

- Reliable estimates of current spending on maternal, newborn, and child health (MNCH)—and of how much additional funding is needed—are a critical precondition for sound policy and decision making.
- The Countdown to 2015 initiative estimates that, in 2006, donors spent US$3.48 billion on MNCH, of which 66% was spent on child health and 34% on maternal and newborn health, but these estimates suffer from several limitations.
- Updated estimates for 2007, released by Countdown in June 2010 but not yet available at the time of writing this article, have not addressed these limitations.
- The Consensus for Maternal, Newborn and Child Health argues that US$30 billion of additional funding is needed to save the lives of over 10 million women and children by 2015.
- This US$30 billion “price tag” is misleadingly low because it leaves out crucial service delivery costs.
- There is an urgent need to improve both the tracking of MNCH financing flows and the estimation of additional MNCH resources required to reach the child and maternal Millennium Development Goals.

The crisis of avoidable maternal, newborn, and child deaths in developing countries is currently a major focus for the global health community (Box 1), and it will be one of the leading issues discussed at the September 2010 Summit on the Millennium Development Goals (MDGs) [1]–[3]. Many countries are off track to reach the 2015 child and maternal health MDGs (MDGs 4 and 5), and additional donor assistance will be needed to help countries get back on track.

**Box 1. The Ongoing Crisis of Maternal, Neonatal, and Newborn Deaths**

*Based on references* *[1]–[3]*

**Maternal mortality:** Between 1990 and 2005, the global maternal mortality ratio fell very little, from 430 to 400 per 100,000 live births.

**Child mortality:** The global under-5 mortality rate declined by 28% between 1990 and 2008. Substantial, but not sufficient, progress has been achieved toward MDG 4.

**Neonatal mortality:** 41% of deaths among children under 5 years occur in the first month of life.

How much donor assistance is currently available for maternal, newborn, and child health (MNCH) and how much additional financing will be needed? In this article, we examine the best estimates of current donor assistance to MNCH and of future funding that will be needed to reach MDGs 4 and 5. We lay out several limitations in these estimates. We end with our recommendations for improving the tracking of MNCH financing flows and estimating the costs of scaling up MNCH interventions.

## Tracking Development Assistance to MNCH

The key source of data for estimating official development assistance (ODA) to MNCH is the Countdown to 2015 Initiative (http://www.countdown2015mnch.org). Countdown is a collaborative network of organizations that monitors coverage levels for interventions proven to reduce maternal, newborn, and child mortality. It published its first two reports in 2005 and 2008 (http://www.countdown2015mnch.org/reports-publications), and two articles in *The Lancet* in 2006 and 2008, that include the MNCH financing estimates (covering the years 2003–2006) [4],[5]. Its third report, available at http://www.countdown2015mnch.org/reports-publications/2010-report, covers financing flows up to 2007.

### Which Donors Does Countdown Assess?

Countdown examines ODA from 22 donor countries, members of the Development Assistance Committee (DAC) within the Organization for Economic Cooperation and Development (OECD). DAC describes itself as the “venue and voice of the world's major bilateral donors” [6]. DAC countries regularly report their assistance to the OECD Creditor Reporting System (CRS) database, an online database of aid activities [7]. Countdown's analysis also includes contributions from the World Bank, UNICEF, the GAVI Alliance, the Global Fund, and the European Commission.

### A Rise in MNCH Financing

Countdown estimates that donor disbursements for MNCH increased by 64% between 2003 and 2006, from US$2.12 billion to $3.48 billion [4],[5]. Countdown separates child health financing from maternal and neonatal health financing (Box 2). Of the $3.48 billion disbursed in 2006, 66% ($2.31 billion) was spent on child and 34% ($1.17 billion) on maternal and neonatal health.

**Box 2. Expenditures on Child Health versus Maternal and Neonatal Health**

*Based on references* *[4]* *and* *[5]*

**Child health expenditures:** Spending on activities whose primary purpose is to restore, improve, and maintain the health of children aged between 1 week and 5 years, including: management of childhood illnesses (e.g., oral rehydration therapy, zinc for diarrhea management, treatment of malaria, case management of pneumonia); immunization; insecticide-treated nets; breastfeeding and counseling; and micronutrient supplementation.

**Maternal and neonatal health expenditures:** Spending on activities whose primary purpose is to restore, improve, and maintain the health of women and their newborns during pregnancy, childbirth, and the early neonatal period, including: antenatal, childbirth, and postnatal care; insecticide-treated nets for pregnant women; anti-malarial intermittent preventive treatment; prevention of vertical transmission of HIV; and preventive and treatment services for the newborn.

### Where the Money Comes From

In 2006, 54% of donor assistance to MNCH was from bilateral agencies, 31% from multilateral financers (World Bank, UNFPA, UNICEF, and the European Commission), and 15% from the Global Fund and GAVI Alliance. The two leading MNCH financers were the World Bank ($725m) and the US government ($692m). World Bank financing to MNCH, however, may be overinflated because up until 2008 the World Bank was the only organization that reported commitments (*not* disbursements) to the CRS database.

### How Assistance Is Channeled

Nearly all donor support (95%) in 2006 went to funding specific health projects, rather than providing health sector support (3%) or general budgetary support (2%). Countdown differentiated between three project types: (i) MNCH-specific projects; (ii) projects that support general health activities and contribute to MNCH through health system improvement; and (iii) disease-specific projects with benefit to MNCH (e.g., an HIV/AIDS program that assists the general population rather than only mothers and children). In 2006, about 51% ($1.67 billion) of project funding went to MNCH-specific projects (Box 3), while 29% went to general health care projects ($0.95 billion) and 20% to disease-specific projects ($0.65 billion).

**Box 3. Breakdown of 2006 Donor Support for MNCH-Specific Projects**

Within the category of MNCH-specific projects, the breakdown of funding was as follows:

- Immunization projects (28%)
- MNCH projects with an unspecified purpose, e.g., the project title simply referred to “improving the health of mothers” (28%)
- Maternal health/safe motherhood (21%)
- Nutrition (13%)
- Child health (8%)
- Other projects (2%)

Over the 2003–2006 time period, several countries experienced sharp fluctuations in aid inflows to MNCH, driven by fluctuations in disbursements of large-scale programs and initiatives (e.g., the World Bank's malaria booster project). Countdown comments that this volatility makes long-term planning difficult, especially for countries heavily dependent on aid [5].

### Was Funding Matched to Burden of Disease?

Between 2003 and 2006, donor assistance to MNCH was only partially based on needs. While countries with higher under-5 mortality received more assistance per child, assistance to maternal and newborn health was not well targeted towards countries with the greatest needs.

## Limitations in Estimating Funding Flows to MNCH

There are several limitations in the estimates of funding flows to MNCH in Countdown's first two studies [4],[5]. These limitations also apply to the estimates in the June 2010 report—Countdown has not been able to address these limitations in its third report (Countdown member, personal communication). Some, but not all, of these limitations have been acknowledged by Countdown [4].

### CRS Database Lacks an MNCH Category

When donors report to the CRS database, they must choose a specific “purpose code” for their projects. CRS has 17 purpose codes for health (e.g., malaria control) but no discrete category for MNCH. This presented a challenge to Countdown in trying to estimate how much donors were spending on MNCH.

How did Countdown try to overcome this problem? First, based on its own classification of MNCH activities, Countdown screened the CRS database for MNCH financing. Projects were reviewed based on the project title and descriptions, and categorized accordingly. For projects that specifically targeted the health of mothers and/or children (“MNCH-specific” projects), such as child immunization, the entire disbursement was included in the MNCH financing estimate. Using project descriptions to estimate MNCH funding has many pitfalls, as acknowledged by Countdown itself: “these descriptions can be vague, poorly translated by the donor, or in languages that had to be translated with online translation services” [5].

Second, a proportion of the funding for disease-specific projects and for integrated funding (flowing through general health care projects and budget support) was included in the estimates of MNCH funding. As these projects are usually aimed at the general population, not just mothers and children, the entire funding cannot be included. Primary health care projects, for example, are aimed at the general population. Countdown therefore created “allocation factors” [4] to calculate the proportion of disease-specific and integrated funding allocated to MNCH. Box 4 gives a worked example of an allocation factor.

**Box 4. Using an Allocation Factor to Calculate Spending on Childhood HIV/AIDS**

To calculate the proportion of total HIV/AIDS funds spent on treatment of HIV-positive children, Countdown started by looking at the *total* amount of donor funding for HIV/AIDS in a particular country. It then used an allocation factor to estimate how much of this total was spent on children. It used country-level estimates of the proportion of the total population with HIV who were under 5 years of age (e.g., if 10% of people living with HIV/AIDS in a country were children under 5 years, 10% of the HIV/AIDS funding would be included in the MNCH financing estimates).

### Allocation Factors Are Based on Weak Data

The quality of allocation factors relies on the quality of the underlying country-specific data, which are often poor. For example, country-level data on the number of malaria cases in children under 5 years is not available for all Countdown countries. So to indicate the proportion of malaria project funds spent on child health in a country, Countdown used *region-specific* data as the basis for the allocation to child health. Countdown argues that it has used the best available data to create the allocation factors (Countdown member, personal communication). However, many of these proxies are based on outdated data sources from studies done in the early 1990s (see web tables 2 and 3 in reference [4]). To give just one example, to estimate the proportion of total project funding for hospital-level health care that was allocated to mothers, neonates, and children, data from 1993 were used [4]. Countdown acknowledges that “there is uncertainty around the allocation factors and assumptions we use to apportion funds” [4].

### Private and Nontraditional Donors Are Not Included

Funding from foundations (e.g., the Bill & Melinda Gates Foundation), nongovernmental organizations (NGOs), and nontraditional donors (e.g., China) are not recorded in the CRS database and are missing in the Countdown calculations. Yet they probably represent a significant source of MNCH funding. Similarly, the Countdown reports contain no information on domestic MNCH funding from low- and middle-income countries. The 2010 Countdown report continues to exclude these data.

### Disbursements to Family Planning Are Not Captured

Countdown's financing estimates do not include disbursements to family planning, even though family planning is crucial to improving women's health. Countdown plans to include family planning disbursements for the 2012 report, though they are still missing from the 2010 report.

## Additional Financing Needs to Reach MNCH Targets: What Is the “Price Tag”?

### The US$30 Billion Price Tag

How much additional funding is needed to reach MDGs 4 and 5? The “price tag” that has gained most traction in global health circles is US$30 billion, an estimate of the additional amount of funding needed between 2009 and 2015 for MNCH. The estimate comes from the Consensus for Maternal, Newborn and Child Health, a statement published by the Partnership for Maternal, Newborn and Child Health (PMNCH), a global alliance of over 300 MNCH organizations [8]. The estimate is based on calculations included in a report by the High-Level Taskforce on Innovative International Financing for Health Systems (the HLTF) [9]. The Taskforce established an independent working group in 2008 to estimate the costs of achieving the health-related MDGs in 49 low-income countries, with a special emphasis on MDGs 4 and 5.

The group established two different technical teams, which developed two separate cost estimates using different methods. One team was led by WHO (with UNAIDS and UNFPA), the other by the World Bank (with UNICEF, PMNCH, and UNFPA). Both teams calculated the additional program costs for eight health programs crucial to reaching the MDGs: immunization, management of child illnesses, maternal health, family planning, TB, malaria, HIV/AIDS, and essential drugs. Program costs included expenditures for drugs, vaccines, and medical supplies; infrastructure costs to overcome program-specific barriers; and program management costs. In addition to the program costs, the teams estimated the costs for providing crosscutting health systems strengthening (HSS), including the training and remuneration of health workers and the building of new clinics.

### What Does the US$30 Billion Pay For?

The $30 billion figure in the MNCH Consensus is based on WHO's estimates of the *program costs alone* between 2009 and 2015 required to scale up key MNCH interventions related to *just four* of the eight programs to universal coverage levels [9]. The US$30 billion provides an additional US$11.82 billion for maternal health, US$8.43 billion for family planning, US$2.53 billion for the management of childhood illnesses, and US$6.27 billion for immunization, adding up to US$29.05 billion. The Consensus does not clarify what the remaining US$0.95 billion would cover.

## Limitations in Estimating the MNCH “Price Tag”

### The Price Tag Leaves Out HIV, Malaria, TB, and Essential Drugs

The US$30 billion program costs do not include the costs of HIV/AIDS, TB, and malaria interventions relevant to MNCH (e.g., drugs to treat children with malaria). Nor do they include the costs to increase access to essential drugs for treating chronic and neglected tropical diseases. The additional costs of including interventions for HIV/AIDS, TB, and malaria and essential drugs specifically for mothers, newborns, and children is unclear from the HLTF report. The report only gives the total figures for these four health programs across the whole population: US$15.13 billion is needed for HIV/AIDS, US$7.25 billion for malaria, US$4.78 billion for TB, and US$9.78 billion to increase access to essential drugs [9],[10]. A substantial proportion of these costs will be relevant to MNCH.

### The Price Tag Leaves Out HSS

A more serious omission is that the price tag does not include HSS, i.e., the costs to scale up the system-wide components, including human resources, which would allow programs to function effectively. According to the WHO estimates for the HLTF, US$185.7 billion is needed for HSS [9],[10]. Again, a substantial proportion of this figure is highly relevant to MDGs 4 and 5.

### The Costing Estimate Is Misleading

The Consensus for Maternal, Newborn and Child Health suggests that US$30 billion will save “the lives of over 10 million women and children by 2015” [8], a suggestion that has gained traction among donors and MNCH advocates. But the US$30 billion alone is unlikely to save over 10 million lives, since it must be complemented by a huge amount of additional funding for human resources and other crosscutting health system components.

A recent UNFPA study estimates that meeting existing needs for family planning and maternal and newborn health *alone* would cost an additional US$12.8 billion *annually* [11]. This estimate—which includes the costs of drugs and supplies, human resource costs, and other health systems costs needed for effective service delivery—indicates that many more resources are required for scaling up MNCH interventions than are stated in the PMNCH Consensus.

### Disagreement about Costing Methods

The MNCH costing work is hampered by disagreement about the best methodology used to estimate the financing needs. For the report by the HLTF, WHO and the World Bank came up with very different figures for the MNCH price tag, in part because they used different methods (Box 5). While the WHO figure on the programmatic costs for MNCH was US$30 billion, the World Bank figure was considerably lower. The World Bank estimate of the additional funding needs for maternal health, family planning, management of child diseases, and immunization was just US$16.97 billion (though it estimates that the cost for crosscutting HSS is US$68.9 billion).

**Box 5. Different Methods Used by the WHO and World Bank to Estimate the MNCH “Price Tag”**

**WHO approach:** WHO uses a *normative costing approach* that estimates the resources required to scale up country health systems to universal coverage levels; i.e., it estimates the cost of meeting the health MDGs by using country-specific intervention costs and then multiplying by the uncovered population. The normative approach considers the amount of resources required to scale up country health systems to a level that is considered “best practice” and responds to the technical requirements for scaling up established by the various technical programs [11].

**World Bank approach:** The World Bank's *marginal budgeting for bottlenecks* approach considerably differs from WHO's costing approach. Building on health data reported by developing countries, it identifies important health systems constraints (bottlenecks) and then calculates the cost of strategies to remove programmatic and health systems bottlenecks, and their returns in terms of health outcomes. For the HLTF report, the World Bank team has calculated three different scaling up scenarios, of which only one (medium) was included in the report [19].

The estimates vary not only because of the underlying methods but also because of diverging views on how to best scale up services to meet the MDGs. World Bank estimates assume a delivery strategy that emphasizes full scale up of community-based services *before* expanding clinical services. Major investments for the provision of clinical services are not introduced until the final years of the period 2009–2015. Its scale-up targets are less ambitious than the WHO's targets. The WHO costs are based on a facility-based approach, emphasizing the building of new health centers and hospitals and the need for more nurses and midwives. The WHO approach takes a more optimistic view of the speed with which new infrastructure can be put into place.

## Policy Recommendations

Below, we offer a set of policy recommendations to overcome weaknesses in tracking MNCH financing flows and in estimating the MNCH “price tag.”

### Improved Reporting of MNCH Financing Data

First, although the timeliness of donor reporting has improved in recent years, there is room for improvement. While OECD donors were expected to report their 2008 financing data to the DAC's Statistics and Monitoring Division by mid-July 2009, only half of them complied with this reporting deadline (DAC Secretariat, personal communication). Many donor governments provided the requested data in October 2009, whereas one major donor only made the data available in December 2009 (i.e., five months late).

Large donors in particular, such as the US and France, with large administrations and many different agencies involved in development finance, often do not report data in a timely way to the CRS. As the DAC only releases the complete yearly data, such reporting behavior by donors delays the timely release of the CRS data. This delay makes it difficult to track if donors are living up to their commitments and contradicts the accountability principles of the Paris Declaration on Aid Effectiveness and the Accra Agenda for Action [12]. Reporting delays are also a stumbling block to answering other important questions in a timely manner, such as how the global economic downturn affects donor assistance to MNCH.

Second, donor countries should better coordinate their MNCH reporting and improve the quality of the reported information. An initial step toward a coordinated reporting format would be for donors to agree upon specified keywords that would be systematically included in the CRS project descriptions. In the context of a WHO request to better track donor assistance to the health MDGs, in 2007 the DAC Secretariat recommended that DAC members use the keyword “child health” (or its equivalent in French) in the project descriptions. However, one member objected to this proposal (DAC Secretariat, personal communication).

Since a key challenge for Countdown is the weak project descriptions in the CRS database, a better use of the project description field by the DAC members is even more important than introducing keywords. Precise, complete, and coherent project descriptions would help to make MNCH financing estimates more evidence-based by showing how funds are spent. This effort should be supported by increased investments in the accounting systems of donors, which are often not designed to track actual MNCH-related disbursements. The main reason why donors are not willing to make better use of the project description field in the CRS database, to introduce keywords that can be used to search for MNCH expenditures, or to improve their accounting systems is the increased reporting costs. However, to achieve better estimates of MNCH financing flows, donors need to invest more in their reporting obligations and accounting systems.

### Better Allocation Factors

The crosscutting nature of MNCH means that simply adding a category called “MNCH” to the CRS database would not be a solution. There will always be to be a need to apportion a percentage of disease-specific and integrated funding to MNCH. Estimates of donor flows to MNCH would be improved through the creation of better allocation factors, which means: (a) updating the data sources used to calculate these factors, and (b) donors investing in the necessary data collection, monitoring, evaluation, and operational research. In the short term, allocation factors that are based on outdated sources should be replaced by factors based on updated data. Donors should fund new studies that help to create better allocation factors (e.g., better data are needed to help estimate the proportion of total project funding for hospital-level health care that gets allocated to mothers, newborns, and children).

### Inclusion of Disbursements from Nontraditional Donors

The contributions of key private financers, such as the Bill & Melinda Gates Foundation, NGOs, and emerging donor governments, can be mined from various sources. We acknowledge that tracking data that fall outside the CRS database presents difficulties, including the increased risk of “double counting” (e.g., Countdown includes funding flows through the GAVI Alliance, but GAVI is itself supported by the Gates Foundation). Yet the Institute for Health Metrics and Evaluation (http://www.healthmetricsandevaluation.org/) has managed to track “nontraditional” funding flows to global health, including funding from foundations and NGOs [13], as well as domestic financing for health [14], suggesting that difficulties in tracking nontraditional funding can be overcome.

### An Updated Price Tag before the September 2010 MDG Summit

More accurate measures of the MNCH price tag are needed—which include health service delivery costs—so that donor governments are given a realistic picture of what it will take to cut maternal, newborn, and child deaths. Accurate estimates are needed to better inform the discussions about MNCH financing at the upcoming Summit on the MDGs in September 2010.

While previous WHO estimates of MNCH funding needs, from 2007, also excluded crosscutting HSS costs, these estimates did at least include important service delivery costs, such as human resource costs [15],[16]. At a minimum, this cost category should be included in the MNCH price tag before the September 2010 MDG Summit.

### Donors Should Support Countries in Using the “One United Nations” Costing Method

Given the different costing methods used by WHO and the World Bank to estimate the MNCH “price tag” (Box 5), we welcome the recent move to create a “one United Nations” costing method for health [17]. An interagency working group—UNAIDS, UNDP, UNFPA, UNICEF, WHO, and the World Bank—is currently harmonizing the various costing tools used in the health sector [17]. The aim is to develop a single UN tool, the Unified Health Model, to support health sector costing, budgeting, financing, and strategy development in developing countries with a focus on medium-term MDG-related health activities. Use of a “one UN” costing tool should contribute to better arguments for an increased commitment to MNCH financing, both domestically and globally, and developing countries should be supported in using the tool.

## Conclusion

Important strategic decisions must be made to accelerate progress toward MDGs 4 and 5. Reliable estimates on the currently available financial resources and the funding gap are a critical precondition for sound decision making and for directing investments.

The current conversations in global health circles about MDGs 4 and 5 refer to the US$30 billion price tag for reaching these goals. By promoting this figure, which omits crucial service delivery costs, we are concerned that the Consensus for Maternal, Newborn and Child Health risks raising false expectations about the funding needed for impact.

There are two things we can say with certainty. First, the current level of aid devoted to MNCH is inadequate, providing only a fraction of the total resources required to achieve the child and maternal health MDGs. Second, donors are not living up to their promises—in 2010, Africa will receive only about US$12 billion of the $25 billion pledged by the G8 at Gleneagles, due largely to the underperformance of several European donors [18]. Scaling up to reach MDGs 4 and 5 means urgently fixing these shortfalls.

## Abbreviations

DAC: Development Assistance Committee
HLTF: High-Level Taskforce on Innovative International Financing for Health Systems
HSS: health systems strengthening
MDG: Millennium Development Goal
MNCH: maternal, newborn, and child health
NGO: nongovernmental organization
ODA: official development assistance
OECD: Organization for Economic Cooperation and Development
PMNCH: Partnership for Maternal, Newborn and Child Health
